# Adalimumab: Another Medication Related to Osteonecrosis of the Jaws?

**DOI:** 10.1155/2016/2856926

**Published:** 2016-03-21

**Authors:** Andrea Cassoni, Umberto Romeo, Valentina Terenzi, Marco Della Monaca, Oriana Rajabtork Zadeh, Ingrid Raponi, Maria Teresa Fadda, Antonella Polimeni, Valentino Valentini

**Affiliations:** Odontostomatological Science and Maxillo facial Surgery Department, “Sapienza” University of Rome, 00161 Rome, Italy

## Abstract

*Objective.* The acronym MRONJ has been created in order to identify “Medication-Related Osteonecrosis of the Jaw,” observed after the use of Bisphosphonates, RANK ligand inhibitor, and antiangiogenic medications. Only a case of osteonecrosis of the jaw in a Chron's disease patient following a course of Bisphosphonate and Adalimumab therapy has been recently described, so that it has been supposed that also this medication could promote manifestation of osteonecrosis.* Clinical Case*. On August, 2014, a 63-year-old female with a history of idiopathic arthritis treated with medical treatment with Adalimumab from 2010 to 2013 presented referring pain in the right mandible.* Results*. This patient presented with nonexposed osteonecrosis of the jaw after placement, on September, 2010, of four titanium fixtures in the mandible.* Conclusions*. The authors suggest that the biologic therapy with an anti-TNF-*α* antibody might promote the manifestation of osteonecrosis and compromise oral healing capacity of the bone.

## 1. Introduction

Osteonecrosis of the jaw related to Bisphosphonate (BRONJ) and, recently, Denosumab related osteonecrosis of the jaws (DRONJ) and other medicaments such as antiangiogenic agents (MRONJ) has been described [1–3]. Adalimumab (Humira®, Human Monoclonal Antibody in Rheumatoid Arthritis) is a human monoclonal TNF-*α* antibody used to threat rheumatoid arthritis, juvenile idiopathic arthritis, psoriatic arthritis, ankylosing spondylitis, plaque psoriasis, and Chron's disease in case of other drugs' failure. Adalimumab binds to tumor necrosis factor-alpha (TNF-*α*). TNF-*α* normally binds to TNF-*α* receptors, which leads to the inflammatory response of autoimmune diseases. By binding to TNF-*α*, Adalimumab reduces this inflammatory response. Common side effects include redness, itching, pain, or swelling at the injection site (it is administered by subcutaneous injection), and because TNF-*α* is part of the immune system that protects the body from infection, treatment with Adalimumab may increase the risk of infections such as tuberculosis, while rarely worsening or initiation of congestive heart failure or of a multiple sclerosis/neurological disease, a lupus-like syndrome, a promotion of lymphoma, and pancytopenia have been reported [4]. Just one case of osteonecrosis of the jaw in a Crohn's disease patient following a course of Bisphosphonate and Adalimumab therapy has been recently described, so that it has been supposed that this medication could promote manifestation of osteonecrosis [5]; to the best of our knowledge, we describe the first case of osteonecrosis of the jaws that could be consequent to the use of Adalimumab to treat idiopathic arthritis in a woman.

## 2. Case Report

**Figure 1:**
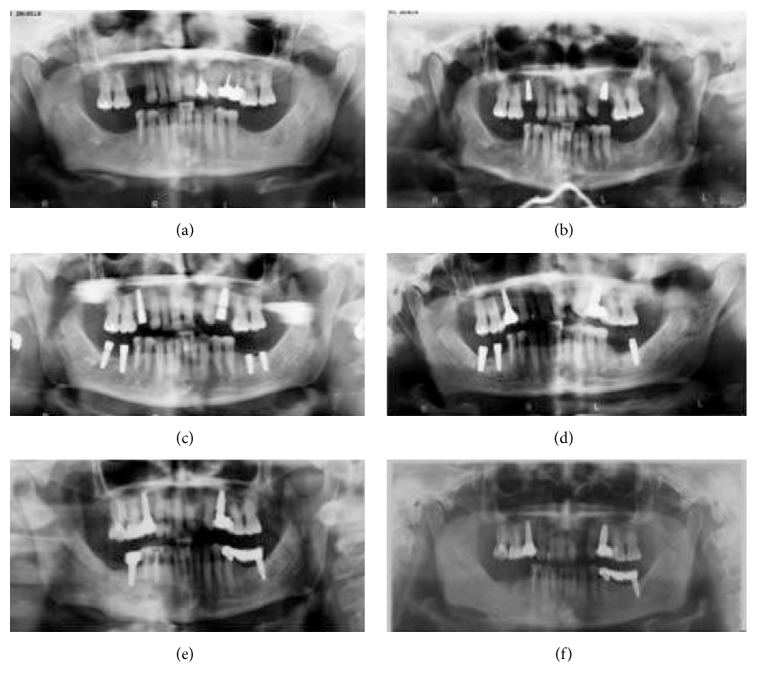
Orthopantomography (OPT) performed on 2009 (a), on May, 2010 (b), on September, 2010 (c), on May, 2011 (d), on August, 2011 (e), and on August, 2014 (f). The last one revealed osteosclerosis, osteolysis, and subperiosteal bone deposition and persistence of extraction socket.

**Figure 2:**
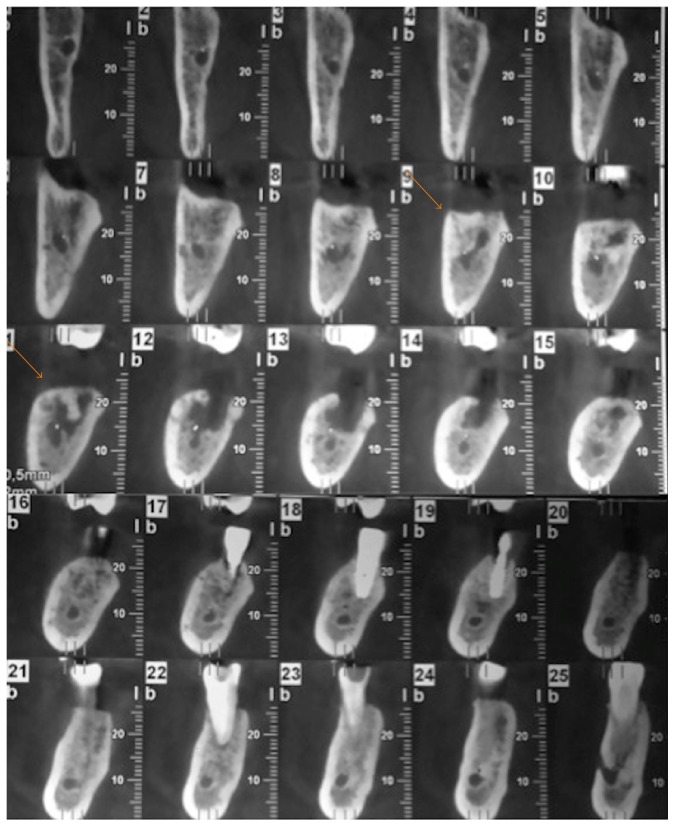
CT Dentascan scan performed on July, 2011, showing sequestrum.

**Figure 3:**
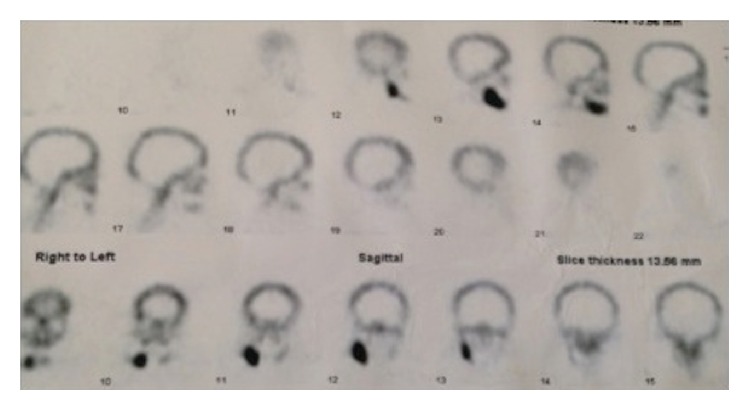
Static whole body bone scintigraphy performed on October, 2013, revealed an intensive trace uptake at the right mandible.

**Figure 4:**
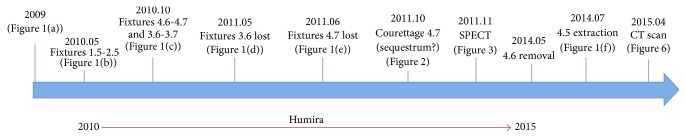
Schematic view of disease progression.

**Figure 5:**
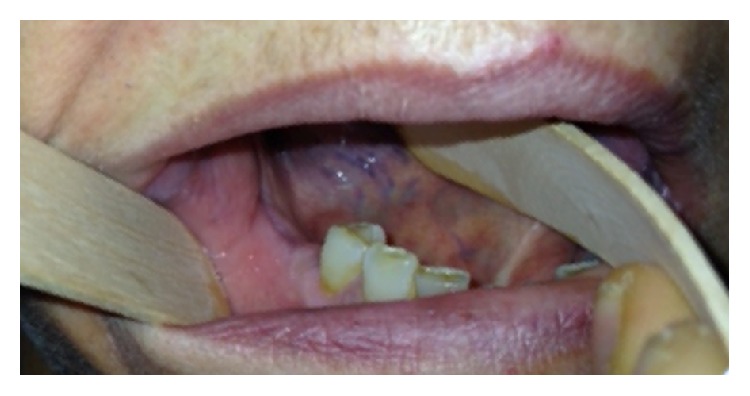
Postoperative control in 2015 showing the healing of the mucosa after extraction of tooth 4.5 and curettage of the bone.

**Figure 6:**
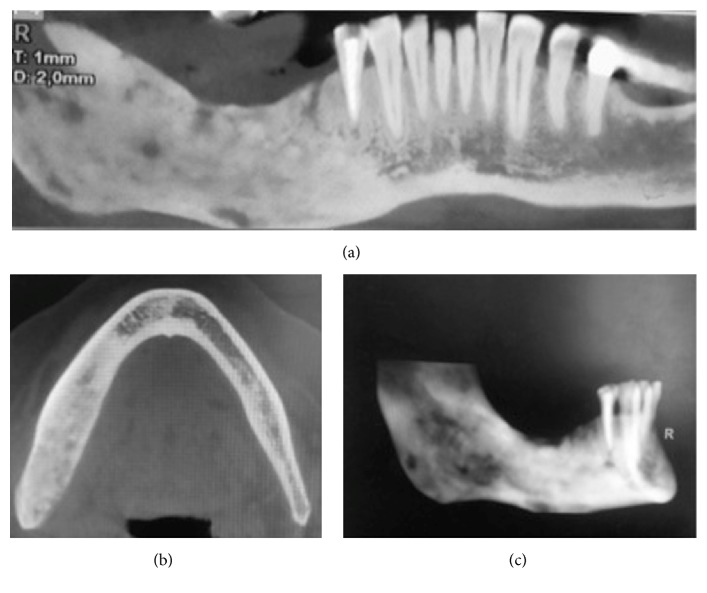
CT scan performed on April, 2015. (a) Panorex view showing sequestrum, (b) axial view, and (c) 3D reconstruction.

A 63-year-old female presented to our center on August 2014 referring pain in the right mandible. No history of smoking or alcohol abuse was referred. Comorbidities include obesity and idiopathic arthritis treated with medical treatment with Salazopyrin on 2009 and with Adalimumab (1 injection every two weeks) from 2010 to 2013: during this period, improvement of symptoms has been referred by the patient. Her physician also proposed a treatment with glucocorticoids, but she refused because of side effects. She referred 4.7 (caries) and 4.8 (impacted tooth) extraction on 2008, before starting any treatment for idiopathic arthritis. An orthopantomography (OPT) performed on 2009, before starting treatment with Humira, did not show bone alterations (Figure 1(a)). On May, 2010, two titanium fixtures were positioned in the maxilla (1.5 and 2.5) (Figure 1(b)) and, on September-October, 2010, four titanium fixtures were put in the mandible by her dentist (3.6, 3.7, 4.6, and 4.7) (Figure 1(c)). On June, 2011, the one positioned in the region of 4.7 was lost, and it was decided to reposition it about 1 month later. On May, 2011, the fixture positioned in the region of 3.6 was lost too (Figure 1(d)), and, moreover, one month later, after appearance of pain in the right mandible, the fixture in the region of 4.7 was removed (Figure 1(e)). A CT scan was performed (Figure 2); because of difficult healing, patient underwent curettage medications with local antibiotics (Rifocin) for about 2-3 months. Patient referred persistence of pain, partially resolved after systemic antibiotic therapy (amoxicillin + clavulanic acid 1 gr twice a day) (Figure 1(e)). A static whole body bone scintigraphy revealed an intensive trace uptake at the right mandible (Figure 3). Nevertheless, no remission of pain was referred and patient underwent systemic therapy with paracetamol and eventually ketorolac in order to control it. Occasionally systemic antibiotic therapy was administered in order to control swelling of the right mandible, and partial resolution of pain has been described. On May, 2014, fixture removal (the one positioned in the site of 4.6) was performed and temporary remission of pain was referred; on July, 2014, due to the persistence of pain, extraction of 4.5 was performed. Orthopantomography revealed osteosclerosis, osteolysis, and subperiosteal bone deposition and persistence of extraction socket (Figure 1(f)). Nevertheless, the patient came to our attention on August 2014 referring recurrence of pain; on the basis of clinical history (Figure 4) and radiological imaging, such as CT scan showing sequestrum, diagnosis of MRONJ with nonexposed necrotic bone was suspected (Figure 5). We considered the patient to be at Stage 0 (nonexposed bone variant) because she presented with no clinical evidence of necrotic bone but presented with nonspecific symptoms and clinical and radiographic findings [1] (odontalgia not explained by an odontogenic cause and aching bone pain in the body of the mandible, loosening of teeth not explained by chronic periodontal disease, alveolar bone loss or resorption not attributable to chronic periodontal disease, changes to trabecular pattern dense woven bone, and persistence of unremodeled bone in extraction sockets and regions of osteosclerosis involving the alveolar bone and/or the surrounding basilar bone) (Figure 6).

The patient reported good pain control using medical therapy consisting in FANS and antibiotics up to March, 2015.

## 3. Discussion

Osteonecrosis of the jaw can be usually observed as an adverse reaction to some medications or to radiotherapy: histologically, it appears similar to osteomyelitis, and it is not clear whether infection is the cause or consequence of bone exposure [6–8]. The term MRONJ has been created in order to identify “Medication-Related Osteonecrosis of the Jaw,” observed after the use of antiresorptive (Denosumab) and antiangiogenic therapies, other than Bisphosphonates [1]. In particular, in the last 2014 AAOMS Position Paper [1], it has been assessed that diagnosis of MRONJ can be done if the following are present:

- Current or previous treatment with antiresorptive or antiangiogenic agents.
- Exposed bone or bone that can be probed through an intraoral or extraoral fistula(e) in the maxillofacial region that has persisted for longer than 8 weeks.
- No history of radiation therapy to the jaws or obvious metastatic disease of the jaws.

Nevertheless, some cases of medication-related ONJ without bone exposure have been described [9]. It is largely reported that BRONJ is caused by apoptosis of osteoclasts, disturbance of osteoclast progenitor cell differentiation and enzyme activity, antineovascularization, and destruction of bone microstructure caused by bone deposition [9, 10]. DRONJ is related to inhibition of the receptor activator of nuclear factor kappa-B ligand (RANKL), resulting in osteoclast function inhibition. Differently from Bisphosphonate, the return of bone turnover to normal can be evidenced about six months after drug injection [2]. The use of glucocorticoids has been demonstrated to lead to osteoblast inhibition and to bone resorption [11]. Also, antiangiogenic medications, such as Bevacizumab and Rituximab, have been found to be implicated in the pathogenesis of ONJ [1, 12, 13].

TNF inhibitors, such as Adalimumab, seem to be able to arrest systemic bone loss assessed by bone mineral density and bone turnover markers in rheumatoid arthritis; nevertheless, evidences of the effect of anti-TNF treatment in preventing fractures are still scarce [14].

It has been described that TNF-*α* has an important effect on systemic bone loss in rheumatoid arthritis, since it enhances osteoclasts activity, differentiation and activation, and osteoblasts production and proliferation resulting in an inhibition on bone turnover markers demonstrated by some studies [14].

Nevertheless, no cases related to the use of other drugs have been described, except one in which the use of Adalimumab has been hypothesized to improve necrosis caused by Bisphosphonates. It has been supposed that anti-TNF-*α* treatment could cause an inhibition of bone turnover mediated by a reduction of RANKL which could already be shown in patients with rheumatoid arthritis and anti-TNF-*α* therapy [5]. Another hypothesis is the fact that the induced apoptosis of activated human monocytes can lead to worsening of bone repair after jaw necrosis. Nevertheless, since one of the side effects described after Adalimumab treatment are infectious complications due to immunosuppression, it can be proposed that osteonecrotic lesions of the jaws can “occur because of spreading ongoing infections” [5, 15].

Local factors related to ONJ are dentoalveolar surgery, denture use, and preexisting inflammatory dental disease [1]. In case of nonexposed Bisphosphonate-related osteonecrosis and asymptomatic patients (no clinical evidence of infection and radiographic findings can be present), only follow-up is advocated [10]; in our case, only pain was referred, and FANS associated with systemic antibiotics were administered in order to control it. A chronic infection was also present, and patient referred multiple local medications to gain healing after fixture removal. Radiological signs include osteosclerosis, cortical disruption, osteolysis, subperiosteal bone deposition, thickening of lamina dura, and widening of periodontal ligament; in addiction, surface irregularity and persistent sockets strictly correlate with ONJ [16, 17]. Osteosclerosis is due to mineralization in the absence of balanced bone resorption, and the persistence of extraction sockets results from marked inhibition of remodeling following removal of the tooth [17]. In our case report, CT scan showed signs of bone sequestrum and analyzing previous OPT signs of osteosclerosis and subperiosteal bone deposition were present. Delayed healing after fixture removal and persistence of pain, associated with these radiological findings and bone scintigraphy, showed positive tracer led us to suspect diagnosis of MRONJ related to Adalimumab [18]. Nevertheless, further case reports are required in order to confirm the hypothesis but this clinical case suggests that a dental exam is indicated before prescription of biological drugs like Adalimumab. In those patients, periodic dental checkup can be recommended too.
